# Modelling binding between CCR5 and CXCR4 receptors and their ligands suggests the surface electrostatic potential of the co-receptor to be a key player in the HIV-1 tropism

**DOI:** 10.1186/1742-4690-10-130

**Published:** 2013-11-11

**Authors:** Olga V Kalinina, Nico Pfeifer, Thomas Lengauer

**Affiliations:** 1 Department for Computational Biology and Applied Algorithmics, Max Planck Institute for Informatics, Campus E1 4, Saarbrücken 66123, Germany

**Keywords:** HIV-1 tropism, Structural model, Coreceptor usage, Entry inhibitor, Tropism test

## Abstract

**Background:**

CCR5 and CXCR4 are the two membrane-standing proteins that, along with CD4, facilitate entry of HIV particles into the host cell. HIV strains differ in their ability to utilize either CCR5 or CXCR4, and this specificity, also known as viral tropism, is largely determined by the sequence of the V3 loop of the viral envelope protein gp120.

**Results:**

With statistical and docking approaches we have computationally analyzed binding preferences of CCR5 and CXCR4 to both V3 loop sequences of virus strains of different tropism and endogenous ligands.

**Conclusions:**

We conclude that the tropism cannot be satisfactorily explained by amino-acid interactions alone, and suggest a two-step mechanism, by which initial coreceptor selection and approach of the ligand to the binding pocket is dominated by charge and glycosylation pattern of the viral envelope.

## Background

To enter a host cell, the HIV envelope protein gp120 binds to the cellular CD4 receptor and to one of the two cellular co-receptors, CCR5 or CXCR4. We call viruses (or their genome sequences) that can bind exclusively to CCR5 R5-tropic, those that can bind exclusively to CXCR4 X4-tropic and those that can bind to either coreceptor dual-tropic viruses. X4 and dual-tropic viruses together form the set of X4-capable viruses. Both CCR5 and CXCR4 belong to a prominent family of G-protein coupled receptors. They bind to chemokines: CCR5 binds to a number of inflammatory CC-chemokines including CCL3, CCL4 and CCL5 [1,2], CXCR4 is a receptor for SDF-1 [3] and extracellular ubiquitin [4]. Viral usage of one or the other coreceptor can vary, and is of pivotal importance for the correct choice of antiviral therapy with a class of drugs called entry inhibitors. The first CCR5 inhibitor, maraviroc, has entered clinical practice in the year 2007 [5], but no inhibitor of CXCR4 is on the market yet. Since an entry inhibitor targeting CCR5 or CXCR4, respectively, can only be effective against viruses with the corresponding tropism, administering entry inhibitors, such as maraviroc, requires an advance test of viral tropism. There are two classes of such tests: phenotypic and genotypic. In phenotypic tests, viral tropism is determined in a laboratory assay. In genotypic tests the viral tropism is inferred from the relevant regions of the viral genome by computational means. Several computational prediction models based on the sequences of a part of the gp120 sequence called the V3 loop have been proposed [6-9]. Even though recently a genotypic test has become available that points to regions in the V3 loop determining tropism [10], neither class of tests provides a mechanistic understanding of the coreceptor choice. In this study, various methods of computational structural biology have been applied to elucidate this mechanism.

To date, only an experimentally resolved structure of nearly full-length gp120 with a short N-terminal peptide of CCR5 [11] is available. This structure has been used recently in a modeling attempt to understand the mechanistic basis of viral tropism [12]. In contrast, we have studied interactions between V3 loop and the full-length co-receptor.

## Results

### Structural analysis of V3 loops corresponding to different virus subtypes does not reveal any tropism preferences

In our study, we used two sets of V3 loop sequences: one set of 7 R5-tropic and 13 X4-capable sequences with experimentally verified tropism [10] and another set of 47 R5-tropic and 49 X4-capable sequences with tropism predicted using geno2pheno-C_NGS-Sanger [8] (see Methods for more details).

**Figure 1:**
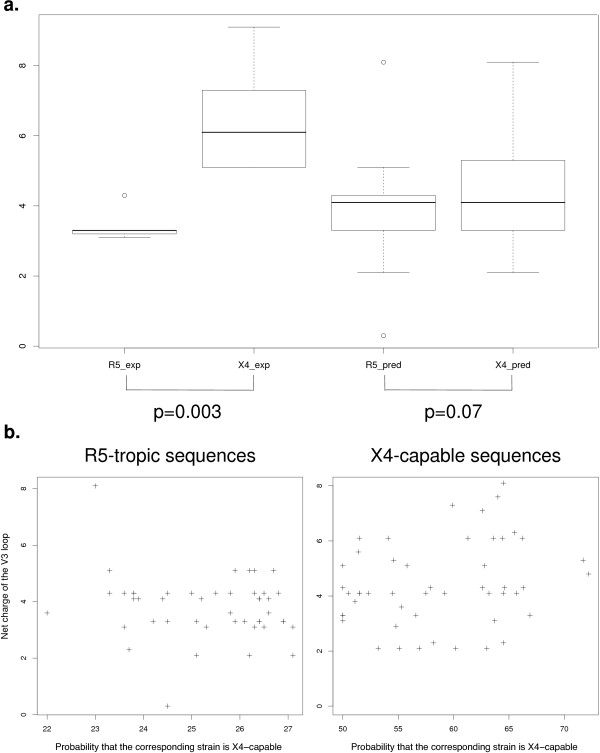
**Charge of V3 loop sequences. a**. Charges of the V3 loops of experimentally tested sequences (exp) and sequences predicted to be prototypic for their tropism (pred). The box plots show median, first and third quartile ±1.5 interquartile range. P-values displayed are based on a two-sided Wilcoxon test. **b**. Dot-plots showing the relationship between the net charge of the V3 loop and the probability of the corresponding strain to show the X4-capable phenotype, as calculated by geno2pheno-C_NGS-Sanger [8], for the set of predicted sequences. P-values based on Pearson’s product–moment correlation test: -0.17 for R5-tropic and 0.25 for X4-capable sequences.

The net charge of V3 loop sequences from the set of experimentally verified cases is significantly higher for X4-capable sequences than for R5-tropic sequences (Figure 1a). However, this may be a consequence of a bias during selection of sequences for experimental testing. The set of sequences with predicted tropism is more diverse and evenly distributed in sequence space than the sequences with experimentally verified tropism: the median and 1st and 3rd quartiles of the pairwise sequence identity are 0.76, 0.71, 0.79 for the predicted R5-tropic; 0.88, 0.88, 0.92 for the experimentally verified R5-tropic; 0.60, 0.68, 0.74 for the predicted X4-capable; and 0.65, 0.71, 0.76 for the experimentally verified X4-capable sequences. The difference of the sequence charge between the two tropism types for the predicted sequences is not very pronounced, although significant, and the median charge is equal for R5-tropic and X4-capable sequences. There is no significant correlation between the net charge of the V3 loop and the probability of exhibiting the X4-capable phenotype, as calculated by geno2pheno-C_NGS-Sanger [8] (p-values -0.17 and 0.25 for R5-tropic and X4-capable sequences, respectively, Figure 1b).

**Figure 2:**
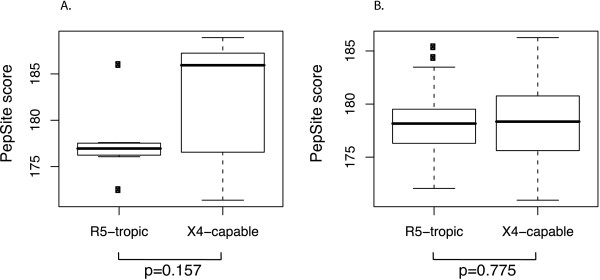
**PepSite scores of V3 loop sequences.** PepSite scores for a 10-amino acid sliding window reflect the propensities of R5-tropic and X4-capable sequences to bind to CXCR4. **A**: experimentally verified sequences; **B**: predicted sequences. P-values displayed are based on a two-sided Wilcoxon test.

PepSite is a computational tool that scans the surface of a given protein for patches that are likely to bind individual amino acid residues or peptides up to ten amino acids [13,14], providing a score that reflects the propensity of the peptide to bind to the protein. The PepSite score is expressed in relative units and the higher scores mean better binding. We apply PepSite in a sliding window of 10 residues to assess the binding of the experimentally verified X4-capable and R5-tropic sequences to CXCR4, and acquire higher average scores for the X4-capable sequences (Figure 2A, low statistical significance here is due to the limited size of the sample). The same procedure for the sequences of the predicted set does not reveal such a trend (Figure 2B). Still, both experimentally verified and predicted X4-capable sequences have significantly higher propensity to bind to CCR5 than to CXCR4 (p-value 0.064 for experimentally tested and 6.156*10^-08^ for predicted sequences based on a two-sided Wilcoxon test). None of the X4-capable sequences has a score for CXCR4 binding that would exceed the score for CCR5 binding by more than 1.5 percentage points. This may indicate that these viruses are equally capable of using both CCR5 and CXCR4 for entry. For R5-tropic sequences, on the contrary, we find a statistically significant higher propensity to bind to CCR5 rather than to CXCR4 in both experimentally verified and predicted sequence sets, in agreement with the assigned phenotype.

**Figure 3:**
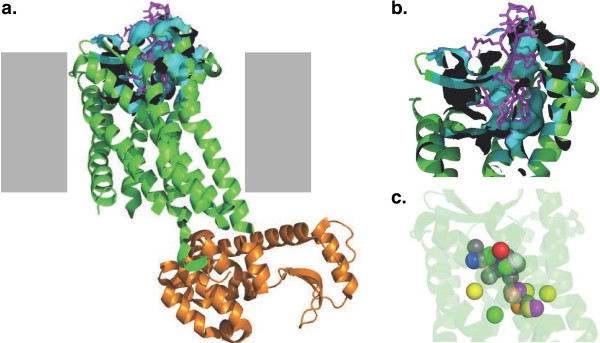
**Structural features of the coreceptor. a**. Overall view of the coreceptor. The CXCR4 polypeptide is shown in green, lysozyme (fused to assist crystallization) is shown in orange, the inhibitor peptide in magenta. The amino acids in contact with the inhibitor peptide are shown in cyan and in surface representation. The approximate position of the membrane is shown as gray rectangles (not in crystal structure). **b**. Cross-section and close-up of the pocket. Colors as in panel a. **c.** PepSite hotspots for amino acid binding on the coreceptor surface. Hotspots for binding of individual amino acids in the pocket of CXCR4 (alanine: salmon; cysteine: cyan; glutamate and aspartate: red; phenylalanine, tryptophan, tyrosine: gray; glycine: orange; histidine: light gray; isoleucine, leucine, valine: yellow; arginine, lysine: blue; methionine: light orange; asparagine, glutamine: green; proline: magenta; serine, threonine: purple). CXCR4 is rendered transparent for clarity.

Both CCR5 and CXCR4 belong to GPCR family of transmembrane proteins and have seven transmembrane helices (Figure 3a). In the crystal structure, CXCR4 is bound to an inhibitor peptide (magenta in Figure 3a) that penetrates deeply into the extracellular pocket of the receptor. The hotspots for individual amino acids are predominantly places inside this pocket, as well. Proline residues are most favorably placed deeply inside the pocket (magenta in Figure 3c), which suggests that the β-hairpin structure of the V3 loop penetrates deeply into the binding pocket of the coreceptor [15]. Aromatic residues are more highly preferred along the channel, and charged residues are situated near the mouth of the pocket (Figure 3c). These preferences are not as pronounced in the CCR5 model as in the structure of CXCR4 (data not shown).

The docking experiment was unable to differentiate between X4-capable and R5-tropic sequences when they were placed into the pocket of CXCR4. The docking energies (FlexPepDock [16] energy scores between -25 and -40 for interface energy and -120 and -280 for whole-complex energy) and the docking poses of the whole loop and its parts do not significantly differ between the sequences of the two tropisms neither in the set of experimentally verified, not in the set of sequences with predicted tropism. Without more experimental information on the precise positioning of the V3 loop in the binding pocket of the coreceptor, it is probably impossible to model this interaction to anything approaching atomic detail. The structural bioinformatics approaches fail to further explain the process of coreceptor binding.

### Structural comparison of the CCR5 and CXCR4 chemokine receptors

CCR5 and CXCR4 are chemokine receptors of the G-protein coupled receptor family, with a global sequence identity of 33%. CXCR4 was recently crystallized as a dimer [15], and it is reported to dimerize in cells independent of ligand binding [17]. The structure forms a large pocket on the extracellular side where the ligand binds. The same pocket is presumably used by the V3 loop of gp120 [15]. The structure of CCR5 has not been experimentally resolved, so we have modeled it using a CXCR4 structure (PDB entry 3oe0) as a template. The model checks out as acceptable when tested with PROCHECK [18].

**Figure 4:**
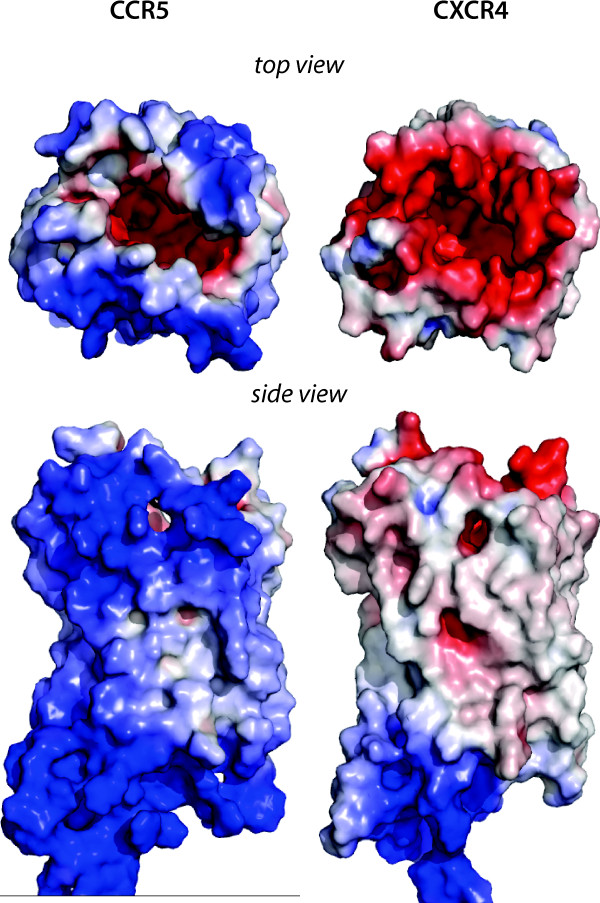
**Surface electrostatic potential of the coreceptors.** Electrostatic potential for structures of CCR5 (modelled) and CXCR4 (modified from PDB entry 3oe0) calculated with ABPS [36]. Red corresponds to a potential of -5 kT/e; blue, 5 kT/e.

Despite their high sequence identity and hence structural similarity, the surface electrostatic potentials of the two receptors differ substantially (Figure 4): the binding pocket of CXCR4 and the area near its mouth are negatively charged, while CCR5 has a negatively charged pocket and a neutral to positive entrance to that pocket. The N-terminus that bears a significant negative charge due to sulfation [19,20] is disordered and absent from both structures.

### Comparison of endogenous ligands

**Figure 5:**
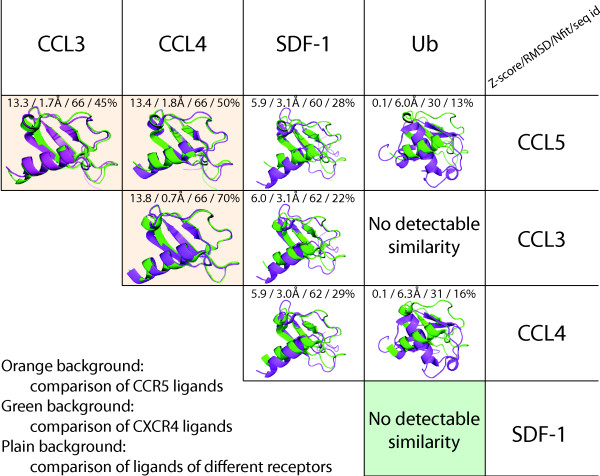
**Structural similarity of endogenous ligands of CCR5 and CXCR4.** Each cell presents a structural alignment of two ligands (one shown in green and the other in magenta; for clarity, only alignment of the first chain of the structures of each ligand is shown). The comparison was performed with DaliLite [21], the obtained Z-scores, RMSD (root-mean-square deviation) of C_α_ atoms, number of aligned residues, and percent of the identical residues in the alignment are reported in each cell.

Both CCR5 and CXCR4 are chemokine receptors. CCR5 binds to a variety of CC-chemokines including CCL5, as well as CCL3 and CCL4 [1,2]. The ligands of CXCR4 are SDF-1 [3] and extracellular ubiquitin [4]. When structurally compared using DaliLite [21], CCL5, CCL4 and CCL3 exhibit significant structural similarity but little, while still detectable, resemblance to the CXCR4 ligand SDF-1 (Figure 5). CXCR4 ligands exhibit no structural similarity among each other. There is no structural similarity between endogenous ligands of CCR5 or CXCR4 and the structure of the V3 loop as it is crystallized in PDB entries 2b4c or 2qad.

**Figure 6:**
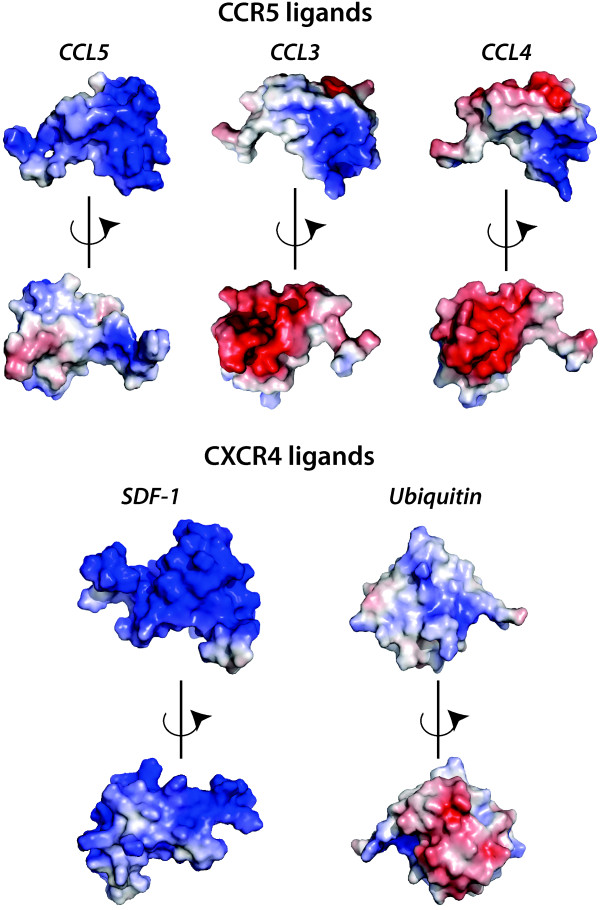
**Surface electrostatic potential of endogenous ligands.** The calculation and the display features are identical to the ones used in Figure 4.

The electrostatic potentials of the ligands are largely compatible with the electrostatic potential of the receptors: CCL3 and CCL4 have negatively charged surfaces, CCL5, although generally positive, is glycosylated [22], hence potentially it can inherit a negative charge from the glycan. SDF-1, in contrast, has a positive potential on the surface, while ubiquitin is generally neutral (Figure 6).

### Analysis of patterns of glycosylation in R5-tropic and X4-capable sequences

We have analyzed the distribution of potential glycosylation sites in R5-tropic and X4-capable sequences. For the dataset of experimentally verified sequences, all R5- and all but two X4-capable sequences contain such a glycosylation site at position 301 (predicted with N-GlycoSite [23]). Of the predicted sequences, all R5-tropic and all but three X4-capable sequences contain this site. This glycosylation site has been proven to be crucial for coreceptor tropism: its removal switches the virus to use CXCR4 [24]. Yet, this site appears to be present in many X4-capable sequences, but it is impossible to assess the extent of its occupancy by computational tools.

**Figure 7:**
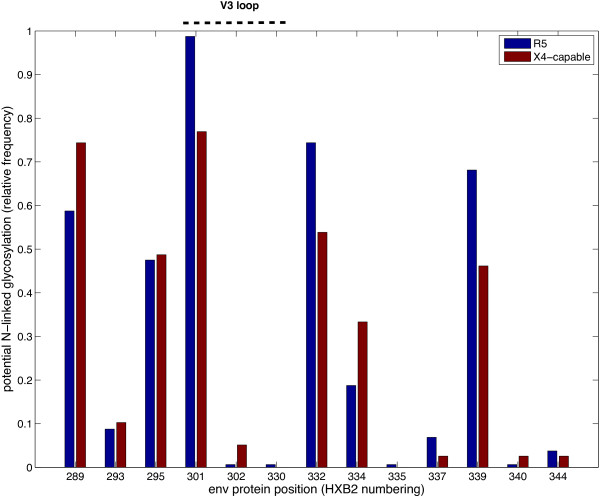
**Predicted glycosylation profile of the V3 loop sequences.** The X-axis shows the positions along the V3 loop (numbering of the whole-length gp120); the Y-axis, the relative frequency of occurrence of a potential glycosylation site (predicted with N-GlycoSite [23]) in the dataset from [25].

There are two other potential glycosylation sites just downstream of the V3 loop at positions 332 and 339. For a larger set of 199 sequences from a study on HIV neutralization by combination of monoclonal anti-bodies [25], R5-tropic strains tend to contain more glycosylation sites at positions 301, 332 and 339 (Figure 7, tropism predicted with geno2pheno [coreceptor] [6] at 10% false positive rate cutoff). For the position 301, the association with tropism type is statistically significant by Fisher’s exact test (p-value 7.64e-06, Bonferroni corrected p-value: 0.0013). Almost all R5-tropic sequences have a glycosylation site at position 301, as opposed to about 80% of X4-capable sequences. Positions 332 and 339 contain a glycosylation site in 70% to 80% of R5- and 50 to 60% of X4-tropic sequences.

## Discussion

We have analyzed binding preferences of the V3 loops of HIV strains of different tropism. PepSite, pursuing a statistical approach, clearly indicates binding of the V3 loop inside the pocket of the co-receptor, but is unable to discriminate between R5- and X4-tropism satisfactorily. Docking also fails to discriminate between the two kinds of tropism. Based on our analysis, we can propose that V3 loops with both kinds of tropism, once placed inside the binding pocket of either coreceptor, would bind comparably tightly.

However, the electrostatics is strikingly different between CXCR4 and CCR5. The electrostatics of their cognate ligands suggests that electrostatic interactions play an important role in the endogenous recognition process [15]. We propose a two-step model of the binding process, in which the first step is characterized by a long-range electrostatic interaction between a coreceptor and its ligand. A suitable potential may preclude the initial contact between the co-receptor and the virus of the non-cognate tropism. This model is consistent with the general two-step model for ligand-receptor recognition, which states that long-range electrostatic interactions govern the formation of non-specific encounter complex, and then binding partners reorient themselves to increase the complementarity of the surfaces [26].

Charge has long been recognized to differ between R5-tropic and X4-capable V3 loop sequences [27], and mutation of neutral to positively charged, or negatively charged to neutral amino acids can switch the virus to using CXCR4 [28]. This may explain the fact that the charge of the V3 loop is a reasonable predictor for virus tropism [29], but not a perfect one. Machine-learning based classifiers improve the prediction [6-9]. Indeed, although the net charge of the V3 loop sequences predicted to be prototypic for their tropism differs significantly between the tropism classes, it cannot account for the entire phenomenon of tropism (Figure 1). We can hypothesize that, although the contribution of the charge is important for the determination of tropism, other factors, such as post-translational modifications, may play a role.

Both CCR5 and CXCR4 are extensively post-translationally modified in their extracellular part; however, most of these modifications reside in a disordered N-terminal region, which is absent from our models. The sulfated acidic N-terminus is characteristic for both proteins [19,20]. The sulfation is critical for interaction between HIV gp120 and CCR5 [30], but not so much for CXCR4 [31]. If the negative N-terminus plays some role in the binding of the more highly conserved base of the V3 loop, which seems plausible by its position on top of the pocket mouth, it can be compensated for by the more negative charge of the pocket entrance of CXCR4. Differential N-glycosylation within the N-terminus of CXCR4 also alters the ability of CXCR4 to bind R5-tropic viruses, but this could not be investigated in the context of the presented study.

As many viral envelope proteins, gp120 is heavily glycosylated, and glycosylation is known to play an important role in viral tropism. Limited evidence in the literature [23,32,33] suggests that R5-tropic viruses tend to be more heavily glycosylated, and the glycans tend to exhibit more complex branching patterns and to be sialylated more frequently, which equips them with negative charge. Removal of a glycosylation site at position 301 leads to an unambiguous switch of tropism from R5 to X4 [24]. Since the exact extent to which every single potential glycosylation site is occupied is not known, structural modeling appears to be inappropriate in this case. However, we observe a statistically significant preference for more potential glycosylation sites in R5-tropic than in X4-capable sequences, position 301 being the most prominent case. Although the trend is pronounced, there remains a large fraction of X4-capable sequences with the glycosylation sites at the positions mentioned above. Not necessarily all these sites are occupied. The known consensus for N-linked glycosylation is rather degenerate (NXT where X is any amino acid except proline). There might be a more complicated, yet unknown, sequence signal that determines glycosylation of 301 N, which assures that R5-tropic sequences are glycosylated more often than X4-capable sequences, or that this glycan is more highly branched and/or more frequently sialylated (which would increase its negative charge) In summary, we hypothesize that differential glycosylation of the HIV envelope gp120 protein is an important factor for choice of co-receptor, and this choice is primarily guided by the V3 loop charge, either defined by the sequences, or conferred by glycosylation.

## Conclusions

We propose a two-step model of interaction between the V3 loop of the HIV Env protein and the CCR5 or CXCR4 coreceptor of the host cell. The choice of the coreceptor is determined at an initial stage by the charge of the V3 loop. We hypothesize that differential glycosylation of HIV envelope gp120 protein is an important factor for choice of co-receptor, as it may alter the charge. This model explains well the available data on the importance of charged residues at certain positions of the V3 loop, as well as the observed glycosylation profile of R5-tropic and X4-capable Env proteins.

## Methods

Experimentally verified V3 loop sequences were retrieved from [10]. As described in [10], viruses were derived from blood samples of HIV-1 infected patients, cultivated in a cell line, and the SI/NSI phenotype was determined by light microscopy. The dataset consists of 7 R5-tropic and 13 X4-tropic sequences.

A set of V3 loop sequences prototypic for each tropism type was obtained with the tropism prediction tool geno2pheno-C_NGS-Sanger [8]. This data set was constructed by predicting coreceptor usage using the coreceptor prediction method introduced in [8] on the Sanger sequences of data from the MOTIVATE as well as the 1029 trial (see [8] for more details on the data set). The sequences were ordered by predicted risk of X4 emergence and the most extreme 47 (risk of X4 emergence smaller than 27.2%) and 49 (risk of X4 emergence larger than or equal to 50%) sequences were selected. The sequences of the second group are predicted to be able to use CXCR4 as the coreceptor (X4-capable), whereas the sequences of the first group are incapable of using CXCR4 and can bind only to CCR5 (R5-tropic). This does not exclude that the sequences in the group of the X4-capable viruses can use CCR5 as well. The new prediction tool geno2pheno-C_NGS-Sanger [8] does not report FPR (false positive rate). Instead the tool outputs a probability that the corresponding virus strain is capable of using CXCR4 (cut-offs mentioned above). In an additional analysis, we predicted the FPR for these sequences using the original tool geno2pheno[coreceptor] and only kept sequences, for which both tools agreed on the tropism call (10% FPR as recommended by the European guidelines [34]). We achieved similar results with this sequence subset (data not shown).

An experimentally resolved three-dimensional structure of CXCR4 (PDB entry 3oe0, [15]) was edited to remove the portion corresponding to T4 lysozyme that was fused to facilitate crystallization. This structure was also used as a template for building a homology model of the CCR5 structure with MODELLER [35] (sequences of CXCR4 and CCR5 have 33% identical and 55% similar amino acids). Preferential peptide localization was calculated using PepSite [14]. Electrostatic potentials were calculated using ABPS [36]. The structural comparison of the endogenous receptor ligands was performed using the DaliLite web server [21].

All sequences of the V3 loop under consideration were modeled onto the structure of the V3 loop extracted from the PDB entry 2b4c by introducing amino acid mutations using FoldX [37]. Only sequences of the same length as in the template structure were used for modeling, hence 17 experimentally verified sequences (7 R5- and 10 X4-tropic) and 72 sequences with predicted tropism (28 R5- and 44 X4-tropic) were modeled. Docking was performed using FlexPepDock protocol [16], a peptide-protein docking protocol with a fully flexible peptide, of the Rosetta modelling suite [38]. The 25 best-scoring modelled structures were considered for each complex. The charge of V3 loops was calculated using Protein Calculator v.3.3 [39]. This tool is not applicable to folded proteins, but due to its high structural flexibility, the V3 loop can be regarded as a flexible peptide.

## Competing interests

The authors declare no competing interests.

## Authors’ contributions

OVK designed the study and performed the experiments. NP designed the study, performed the experiments and the statistical analysis. TL contributed ideas to the design and interpretation of the study. All authors wrote and approved the manuscript.

## Note added in proof

While this manuscript was under peer review, an X-ray structure of human CCR5 in complex with maraviroc was resolved at 2.7 Å [40] (PDB id 4MBS). It supports the model proposed here in several respects:

(i) The structure of the protein is very similar to our model (1.15 Å all atom RMSD over 278 amino acids, which covers the whole CCR5 protein except the inserted rubredoxin).

(ii) The electrostatic surface potential is also observed to differ between CXCR4 and CCR5 [40], thus supporting our two-step binding model.

(iii) The position of maraviroc in the extracellular pocket coincides with the position of the tip of the V3 loop proposed here.
